# A Verification

**Published:** 1889-06

**Authors:** E. A. Ballard


### A VERIFICATION.

In Gregg's illustrated repertory, one of the pains of Kreosotum is shown as starting at the centre of the sternum, extending to left shoulder and down arm. During the last weeks of his life the late Dr. Geo. F. Foster, who died of valvular disease of the heart, suffered a good deal with this pain on both sides, worse on the right. One dose of Kreosote (so-called MM), cured it, leaving the Doctor free from pain during his last days.

E. A. Ballard.

