# Errata

**Published:** 1889-07

**Authors:** 


Errata.—June number, page 259, under the heading "A Verification," for "left shoulder and down arm" read *right shoulder*, etc.

For "Dr. George F. Foster" read *Dr. George F. Foote*, a palpable error.

